# Leveraging Social Media for Population Health Promotion: Evaluation of a Year-Long Nonprofit Public Health Campaign

**DOI:** 10.7759/cureus.105854

**Published:** 2026-03-25

**Authors:** Rohan M Patel, Nicole Cohen, Michael De Lucca, Claudia A Serna

**Affiliations:** 1 Medicine, Nova Southeastern University Dr. Kiran C. Patel College of Osteopathic Medicine, Fort Lauderdale, USA; 2 Epidemiology and Public Health, Public Information Office, Broward Regional Health Planning Council, Hollywood, USA; 3 Community Affairs, Broward Regional Health Planning Council, Hollywood, USA; 4 Public Health, Nova Southeastern University, Davie, USA

**Keywords:** health literacy, health planning, non-profit organization, preventive health, public health, public health program development, social determinants of health, social media, social media analytics

## Abstract

In an ever-evolving health communication landscape, nonprofit organizations must move beyond just maintaining a passive digital presence. Rather, they must actively engage communities to promote meaningful health outcomes. The Broward Regional Health Planning Council (BRHPC), serving Broward County, Florida, executed a year-long public health social media campaign to increase awareness of and educate the community about preventable health conditions. Among these were cardiovascular disease, oral cancer, and obesity. The campaign targeted Broward County’s diverse audience of community residents, healthcare providers, and community partners. Using Facebook, Instagram, X, and LinkedIn, BRHPC disseminated health education information and encouraged online community interaction. In collaboration with the Nova Southeastern University Dr. Kiran C. Patel College of Osteopathic Medicine’s Public Health department, undergraduate and graduate students contributed to the generation of monthly educational content and assisted in evaluating key campaign performance metrics. Key performance indicators included follower growth, engagement (likes, comments, and shares), and reach/impressions. The campaign employed a combination of non-boosted (organic) and boosted (paid) posts over 12 months, gaining a total reach of 151,134 and total engagement of 44,393 across the various social media platforms utilized. These metrics demonstrate significant visibility and audience interaction with the campaign’s public health messaging. This initiative illustrates how strategic social media engagement can expand the reach of public health communication efforts and increase community interaction through educational content. The campaign highlights the potential for nonprofit organizations to leverage digital platforms, strategic planning, and academic partnerships to disseminate health information and promote community awareness of preventable health conditions. Although significant results were seen, a limitation of this evaluation, and potential future research area, is that the engagement metrics did not directly measure whether exposure to the campaign resulted in legitimate health behavior change among the target audience.

## Introduction

In the digital era, social media has transformed the way public health organizations communicate, engage, and mobilize communities toward healthier behaviors. Platforms such as Facebook, Instagram, LinkedIn, and X enable rapid information dissemination, interactive engagement, and real-time feedback. These features are increasingly critical for addressing chronic-disease prevention, mental-health promotion, and health equity [1,2]. The widespread adoption of mobile technology has made digital engagement both feasible and essential for reaching diverse populations; if utilized comprehensively, social media can even tailor health messages to local population needs [3].

Theoretical frameworks from health communication exemplify how social media campaigns can influence behavior. The Social Cognitive Theory posits that behavior change arises through observational learning and social reinforcement, processes enhanced when audiences encounter relatable peers modeling preventive behaviors online [4]. Similarly, the Health Belief Model suggests that messages emphasizing susceptibility, severity, and self-efficacy can motivate preventive action [5]. The Diffusion of Innovations Theory explains how trusted leaders and peer networks facilitate the spread of new ideas through digital sharing [6]. 

Nonprofit organizations are uniquely positioned within these public health frameworks. They often enjoy strong community trust but operate with limited marketing budgets, making cost-effective digital outreach pivotal to their long-term missions. Research shows that nonprofits struggle to sustain engagement yet can achieve deep relational communication when they post interactively and authentically [7]. Paid, or “boosted,” content can enhance visibility within algorithmic feeds, whereas organic posts build credibility and long-term connections [8]. Moreover, academic partnerships strengthen the rigor and reach of such initiatives, ensuring evidence-based content and meaningful evaluation [9,10].

With this, the Broward Regional Health Planning Council (BRHPC), serving Broward County, Florida, launched a year-long digital public health awareness campaign from February 2024 to February 2025, in partnership with Nova Southeastern University’s Dr. Kiran C. Patel College of Osteopathic Medicine’s Department of Public Health (NSU). The campaign sought to increase awareness of preventable conditions, including cardiovascular disease, oral cancer, and obesity, while strengthening local health literacy and community engagement. This paper describes the campaign’s design, implementation, and outcomes, examining reach and engagement across themes and platforms. Framed within established public health communication theories and the literature on digital public health practice, our analysis offers a model for nonprofit-academic collaborations that maximize engagement through strategic, data-driven social media planning. This study evaluates the reach, engagement, and implementation of a year-long strategic public health social media campaign conducted by the BRHPC and NSU to promote awareness and community education of preventable health conditions.

## Materials and methods

This project involved a retrospective, descriptive design to evaluate a one-year-long social media health awareness campaign conducted from February 2024 to February 2025 by the BRHPC in partnership with NSU. The campaign followed a calendar of monthly public health observances coupled with national awareness initiatives (Table 1). The observances were chosen from the Society for Public Health Education's (SOPHE) focus areas [11]. For the months with numerous SOPHE health initiatives listed, students picked the topics that they wanted to generate educational posts for based on individual interest. Among these were Heart Health Month, Oral Cancer Awareness Month, Childhood Obesity Awareness Month, and Kidney Awareness Month, among others. For each observance, messages, visuals, and hashtags were created to emphasize prevention, screening, and lifestyle modifications that members of the community could adopt. Information within these generated materials was procured from reputable health agencies, including the Centers for Disease Control (CDC) and National Institutes of Health (NIH). The posts were developed by undergraduate students in the Bachelor of Science in Public Health (BPH) program and graduate students in the Master of Public Health (MPH) program; they had to be enrolled in the Independent Study course at NSU. These students collaborated with BRHPC’s Public Information Office to research the topics, design posts, and schedule post publication, merging academic rigor with creative practice. 

**Table 1:** Public Health observances & National Health Initiatives by month Each month had between one and four public health observances. If 'N/A' is listed, there were fewer than four observances that month.

Monthly Public Health Themes				
Month	Public Health Theme #1	Public Health Theme #2	Public Health Theme #3	Public Health Theme #4
February 2024	Heart Health Awareness Month	N/A	N/A	N/A
March 2024	Colorectal Cancer Awareness Month	Nutrition Awareness Month	Kidney Health Awareness Month	N/A
April 2024	Oral Cancer Awareness Month	Stress Awareness Month	N/A	N/A
July 2024	Firework Safety Awareness Month	National Grilling Month	N/A	N/A
August 2024	Hurricane Preparedness Month	Immunization Awareness Month	N/A	N/A
September 2024	Childhood Obesity Awareness Month	Healthy Aging Month	N/A	N/A
October 2024	Dental Hygiene Month	Mental Health Tips Month	Breast Cancer Awareness Month	ADHD Awareness Month
November 2024	Lung Cancer Awareness Month	Diabetes Awareness Month	Epilepsy Awareness Month	Healthy Skin Awareness Month
December 2024	Seasonal Affective Disorder Awareness Month	HIV/AIDS Awareness Month	N/A	N/A
January 2025	Thyroid Awareness Month	Cervical Cancer Awareness Month	Birth Defects Awareness Month	Blood Donor Awareness Month
February 2025	Gum Disease Awareness Month	Children's Dental Health Month	N/A	N/A

**Figure 1:**
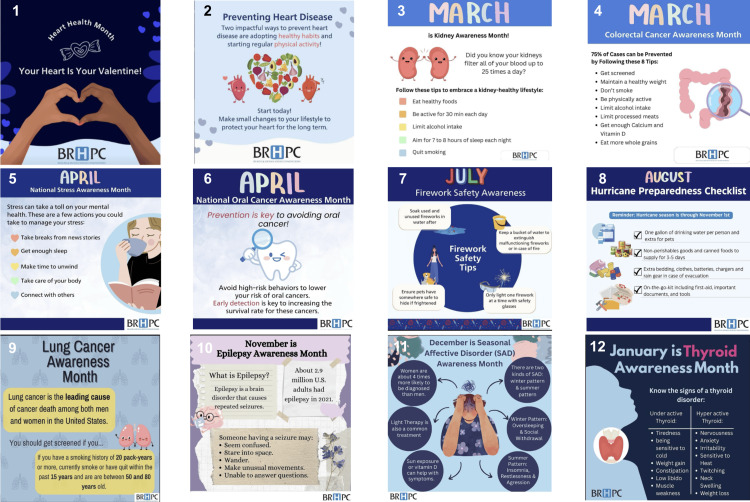
Examples of social media posts, in accordance with monthly themes and public health observances Note:not all posted images for each monthly theme were included 1 and 2: Heart health awareness month (February 2024) 3: Kidney disease awareness month (March 2024) 4: Colorectal cancer awareness month (March 2024) 5: Stress awareness month (April 2024) 6: Oral cancer awareness month (April 2024) 7: Firework safety awareness month (July 2024) 8: Hurricane preparedness month (August 2024) 9: Lung cancer awareness month (November 2024) 10: Epilepsy awareness month (November 2024) 11: Seasonal affective disorder awareness month (December 2024) 12: Thyroid awareness month (January 2025)

Four primary platforms were used: Facebook (both boosted and organic), Instagram, LinkedIn, and X. Each platform was provided with the same infographic or flyer with captions that included relevant hashtags (Figure 1). Monthly analytics were extracted from each platform’s insights dashboard. Key performance indicators included reach (defined as users who viewed content), engagement (measured by aggregate likes, comments, shares, clicks, and profile visits), and likes, which were representative of baseline audience engagement. Facebook posts were categorized as boosted, meaning a payment was provided to increase visibility, or non-boosted. All other social media platforms used analyzed non-boosted data. Data were grouped by month, theme, and platform to assess longitudinal trends. 

Although multiple posts were generated for the same observance at times, a majority of observances had only one post generated for it (Table 2). Descriptive analysis summarized total reach and engagement, while comparative evaluation of boosted versus non-boosted posts assessed relative visibility. There was no pre-determined, mandated posting frequency; BRHPC reserved the right to post the generated graphics as they saw fit within the calendar month, based on the organization's posting schedule. All data used was aggregated from platform-specific user-accessible engagement metrics. Thus, this evaluation did not involve human subjects nor require institutional review board (IRB) review. 

**Table 2:** Number of individual posts generated for each public health observance/theme

Month	Public Health Observance/Theme	Number of Posts Generated
February 2024	Heart Health Awareness Month	4
March 2024	Colorectal Cancer Awareness Month	1
March 2024	Nutrition Awareness Month	1
March 2024	Kidney Health Awareness Month	1
April 2024	Oral Cancer Awareness Month	2
April 2024	Stress Awareness Month	2
July 2024	Firework Safety Awareness Month	2
July 2024	National Grilling Month	1
August 2024	Hurricane Preparedness Month	1
August 2024	Immunization Awareness Month	1
September 2024	Childhood Obesity Awareness Month	1
September 2024	Healthy Aging Month	1
October 2024	Dental Hygiene Month	1
October 2024	Mental Health Tips Month	1
October 2024	Breast Cancer Awareness Month	1
October 2024	ADHD Awareness Month	1
November 2024	Lung Cancer Awareness Month	1
November 2024	Diabetes Awareness Month	1
November 2024	Epilepsy Awareness Month	1
November 2024	Healthy Skin Awareness Month	1
December 2024	Seasonal Affective Disorder Month	1
December 2024	HIV/AIDS Awareness Month	1
January 2025	Thyroid Awareness Month	1
January 2025	Cervical Cancer Awareness Month	1
January 2025	Birth Defects Awareness Month	1
January 2025	Blood Donor Awareness Month	1
February 2025	Gum Disease Awareness Month	1
February 2025	Children's Dental Health Month	1

## Results

**Figure 2:**
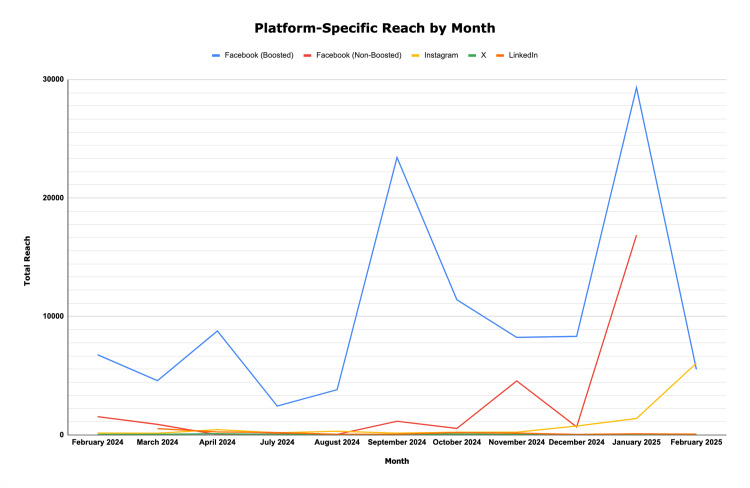
Platform-specific reach by month

**Figure 3:**
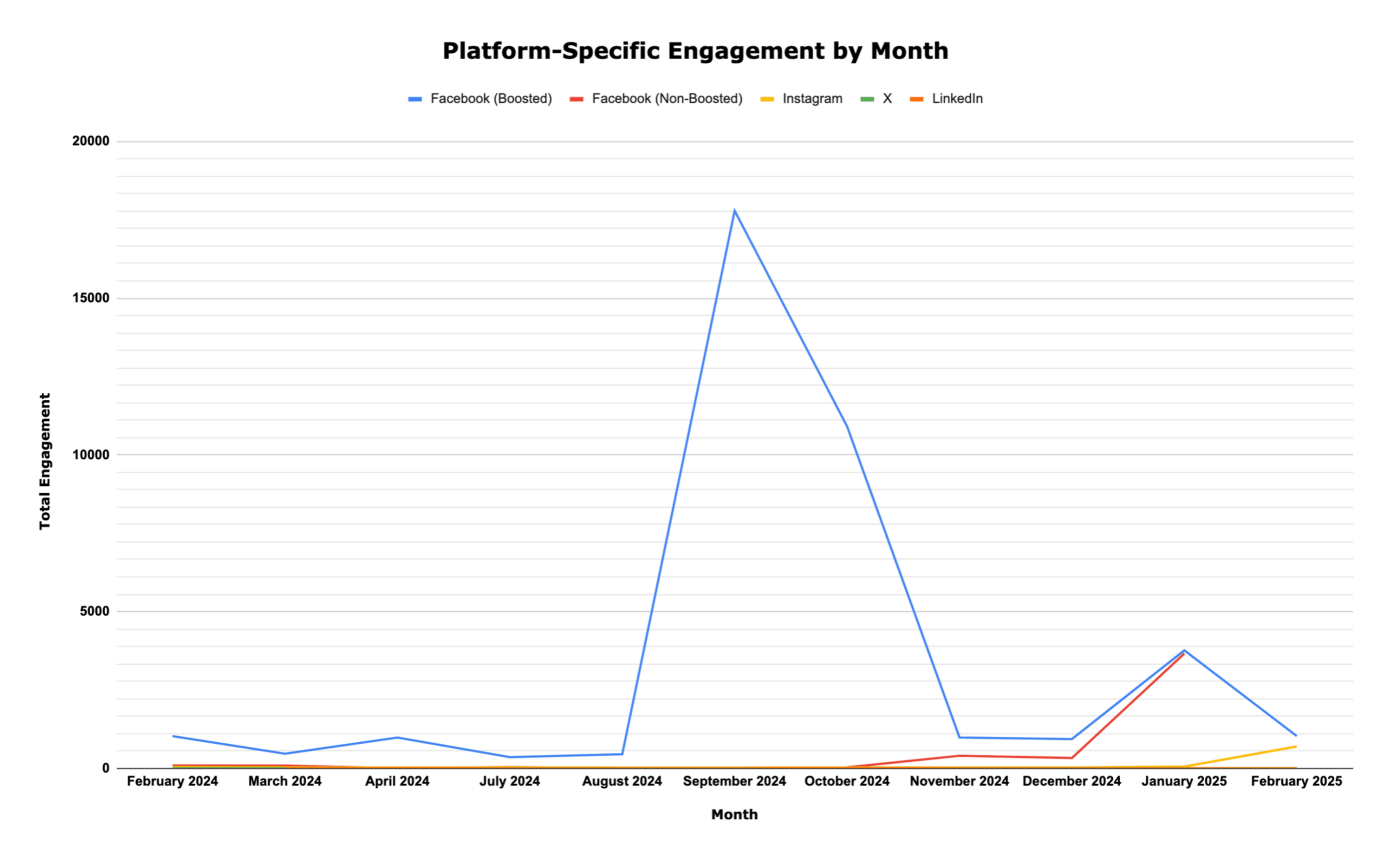
Platform-specific engagement by month

Across all platforms, the campaign achieved a total reach of 151,134 and total engagement of 44,393 (Table 3). Facebook generated the largest share of activity, with boosted posts significantly outperforming organic content (Figure 2 and Figure 3). Paid promotion routinely expanded visibility to audiences exceeding 10,000 users per month, while comparable non-boosted posts reached in the hundreds or low thousands. For instance, during Healthy Aging Month (September 2024), cumulative metrics from all the posts surrounding this topic, including boosted Facebook posts, generated a reach of 13,828 and 9,528 engagements, the highest monthly totals observed. The same post, without Facebook boosting, attained a reach of 573 and an engagement of six. Nevertheless, non-boosted posts, too, demonstrated a notable impact when message relevance was high. During Lung Cancer Awareness Month (November 2024), posts reached 4,229 users and achieved 366 engagements without boosted amplification, reflecting strong resonance with community priorities. The added boost on Facebook resulted in 8,228 reaches. Early campaigns such as Heart Health Awareness Month (February 2024) also garnered moderate engagement, with a reach of 1,746 and engagement of 344, illustrating progressive momentum growth across the campaign timeline.

**Table 3:** Social media analytics report by cumulative monthly metrics (note: some months featured multiple posts for the same public health theme; this table represents the cumulative numbers from each of those posts) ‘N/A’ signifies that analytic data was not available for that post

Social Media Reach and Engagements By Month and Platform												
Month	Reach: Facebook (Boosted)	Engagement: Facebook (Boosted)	Reach: Facebook (Non-Boosted)	Engagement: Facebook (Non-Boosted)	Reach: Instagram	Engagement: Instagram	Reach: X	Engagement: X	Reach: LinkedIn	Engagement: LinkedIn	Total Reach	Total Engagement
February 2024	6,765	1,028	1,540	91	152	43	30	0	n/a	n/a	8,487	1,162
March 2024	4,577	466	887	86	139	33	45	0	530	9	6,178	594
April 2024	8,775	981	27	1	445	27	99	0	252	11	9,598	1,020
July 2024	2,433	357	91	37	176	30	43	0	196	3	2,939	427
August 2024	3,816	448	41	8	311	22	32	0	51	1	4,251	479
September 2024	23,421	17,793	1,151	12	144	15	35	0	64	3	24,815	17,823
October 2024	11,408	10,911	545	30	230	26	68	0	197	11	12,448	10,978
November 2024	8,228	982	4,558	401	229	26	43	0	151	3	13,209	1,412
December 2024	8,318	932	677	328	750	27	39	0	38	1	9,822	1,288
January 2025	29,315	3,764	16,864	3,663	1,379	55	54	0	94	2	47,706	7,484
February 2025	5,534	1,028	n/a	n/a	6,058	696	29	0	60	2	11,681	1,726
Total	112,590	38,690	26,381	4,657	10,013	1,000	517	0	1,633	46	151,134	44,393

Seasonal patterns paralleled national public health awareness campaigns. Engagement peaked during widely recognized campaigns such as Breast Cancer Awareness Month (October 2024) and HIV/AIDS Awareness Month (December 2024), suggesting that synchronization with national themes enhanced visibility, likely due to familiarity with topics. Platform analysis revealed that Facebook dominated total engagement, Instagram performed well for visually driven health content, LinkedIn facilitated professional networking in relevant communities, and X contributed minimally. Engagement patterns continued to strengthen into early 2025. During Thyroid Awareness Month (January 2025), the campaign recorded one of its highest overall performances, with a combined reach of 32,889 and 5,575 engagements across Facebook, Instagram, X, and LinkedIn. This surge likely reflected both the relevance of endocrine content and the cumulative audience growth achieved throughout the previous months. The visibility of Thyroid Awareness Month posts also demonstrated how audience familiarity, accumulated followers, and cross-platform reinforcement can produce compounding effects in later phases of the campaign. 

Overall, the data indicated that combining consistent organic posting with selective paid promotion was most effective for sustaining audience growth and maximizing message reach.

## Discussion

The findings from this year-long evaluation highlight how structured, data-driven social media strategies can significantly advance public health communication in nonprofit contexts. The marked difference between boosted and organic posts reinforces evidence that strategic paid promotion improves message visibility within algorithm-driven feeds [12,13]. However, sustained engagement observed for several non-boosted campaigns highlights the continued importance of authentic and locally resonant content, a factor repeatedly emphasized in digital-engagement studies [14]. The collaboration between BRHPC and NSU demonstrates how shared capacity can strengthen both practice and community education efforts. Similar partnerships have demonstrated improved accuracy of health messaging, increased campaign reach, and expanded student skill development in real-world communication analytics [15]. Embedding student teams within nonprofit initiatives can also foster sustainable workforce pipelines for digital public health roles while reducing costs [3].

Beyond the support from theoretical public health doctrines, emerging research demonstrates that digital health campaigns can shape intermediate outcomes such as knowledge, attitudes, and intent to change; sustained behavioral modification, however, often requires multifaceted reinforcement [16]. Integrating social-media outreach with on-the-ground community involvement would likely offer the most effective route to long-term impact. The high reach achieved during Thyroid Awareness Month and Healthy Aging Month exemplifies how cumulative exposure across time can compound visibility, findings consistent with longitudinal engagement models that stress continuity when it comes to social media posting [17].

A key limitation of this study is that engagement metrics measure visibility and interaction rather than true behavior change, echoing concerns raised in previously published public health literature [18]. Future research should employ a mixed-methods design, combining analytics with post-campaign surveys or questionnaires to confirm behavior changes and examine eventual outcomes. Another limitation was that prior to post-design, an evaluation of evidence-based suggestions on which type of post does best per platform was not conducted. In the future, taking a platform-specific approach to post design will be beneficial for optimizing reach and engagement metrics. 

Collectively, the BRHPC-NSU campaign contributes to the growing evidence that nonprofit organizations, when guided by behavioral theory and supported by academic collaboration, can leverage social media to promote health awareness, prompt dialogue, and further build community trust. These results reaffirm that digital communication, when grounded in science and sustained through community partnership, remains a cost-effective and scalable mechanism for improving population health literacy and overall health promotion. 

## Conclusions

This year-long digital public health campaign demonstrates that nonprofit organizations can effectively leverage social media to expand community awareness and engagement. Through careful planning, theoretical grounding, and academic collaboration, BRHPC achieved substantial community engagement on major public health issues. The initiative offers a replicable framework for other public health agencies seeking to balance authenticity with reach in the digital environment. Future campaigns should incorporate demographic targeting, message testing, and behavioral outcome tracking to strengthen evidence pertaining to the impact social media can have on health promotion. As social media continues to shape health communication, data-guided collaborations such as this one will remain essential for improving population health literacy and fostering sustained community trust while influencing downstream population health outcomes.
